# Chronic Inflammation: A Multidisciplinary Analysis of Shared Pathways in Autoimmune, Infectious, and Degenerative Diseases

**DOI:** 10.7759/cureus.82579

**Published:** 2025-04-19

**Authors:** Ayadi Yacine, Muhammad Zain Ali, Amal Bayen Alharbi, Huda Qubayl Alanaz, Ahmad Saud Alrahili, Ahmad A Alkhdairi

**Affiliations:** 1 Department of Intensive Care, Faculty of Medicine, University of Algiers, Algiers, DZA; 2 Department of Medicine, Hunan Normal University, Changsha, CHN; 3 Department of Medicine, Faculty of Medicine, Qassim University, Qassim, SAU; 4 Department of Family Medicine, Royal Saudi Land Forces, Al-Madinah Al-Munawarah, SAU; 5 Department of Family Medicine, Faculty of Medicine, Qassim University, Qassim, SAU

**Keywords:** autoimmune diseases, chronic inflammation, chronic pain, infectious agents, inflammatory biomarkers, narrative review, neuropathy

## Abstract

Chronic inflammation is a prolonged immune response that contributes to various diseases, including neuropathies, autoimmune conditions, and metabolic disorders. This review explores the role of chronic inflammation across multiple medical specialties, emphasizing its impact on diseases such as chronic inflammatory demyelinating polyneuropathy (CIDP), rheumatoid arthritis (RA), and chronic hepatitis. It highlights common inflammatory biomarkers such as tumor necrosis factor-alpha (TNF-α), interleukin-1 beta (IL-1β), and C-reactive protein (CRP), which drive tissue damage and disease progression. Infections, particularly viral and bacterial agents, trigger inflammatory responses through mechanisms such as molecular mimicry and bystander activation, leading to chronic pain and neuropathy. While current treatments, including corticosteroids and biologics, offer some relief, they are often limited by side effects and insufficient symptom resolution. This review suggests that future therapeutic strategies should focus on targeted inflammation modulation, bioelectronic medicine, and early intervention. Addressing the multifaceted nature of chronic inflammatory syndromes is crucial for improving patient outcomes and quality of life.

## Introduction and background

Chronic inflammation is a prolonged, dysregulated immune response that can lead to tissue damage and contribute to the pathogenesis of various diseases. Unlike acute inflammation, which serves as a protective mechanism against infections and injury, chronic inflammation persists beyond the necessary healing phase, often resulting in autoimmune, infectious, and degenerative conditions [1]. In the context of neuropathy and neurophysiology, chronic inflammation plays a central role in diseases such as chronic inflammatory demyelinating polyneuropathy (CIDP) and multifocal motor neuropathy (MMN), which are characterized by immune-mediated damage to peripheral muscle weakness, fatigue, and pain, even with immune-modulating therapies. Electrophysiological studies reveal that axonal loss and delayed treatment initiation significantly influence long-term outcomes, highlighting the importance of early intervention. Chronic pain and fatigue are common comorbidities, further complicating patient management and overall quality of life [2].

Beyond neurophysiology, this inflammation is implicated in numerous other medical specialties. In resuscitation medicine, excessive inflammation is linked with systemic inflammatory response syndrome (SIRS) and sepsis, leading to multiple organ failure. In rheumatology, diseases such as rheumatoid arthritis (RA) are driven by persistent inflammation that leads to joint damage and loss of function, with an estimated global prevalence of 0.5%-1%. In hepatology, conditions such as chronic hepatitis contribute to liver fibrosis and cirrhosis, caused by sustained inflammation [3]. Oral health also reflects the impact of chronic inflammation, as periodontal disease (PD) progresses from gingivitis to periodontitis, driven by increased immune response to bacterial colonization. Furthermore, chronic inflammation in metabolic diseases such as type 2 diabetes mellitus (T2DM) and obesity is known to impair insulin sensitivity and promote systemic inflammation, leading to cardiovascular complications [4].

Across these diseases, common inflammatory biomarkers, such as tumor necrosis factor-alpha (TNF-α), interleukins (interleukin-1 beta (IL-1β) and interleukin-6 (IL-6)), and C-reactive protein (CRP), have been identified, suggesting shared inflammatory pathways that drive tissue damage and disease progression [5]. However, despite substantial advances in understanding the molecular mechanisms of chronic inflammation, there remains a significant gap in effective, targeted therapies. Current treatments primarily aim at modulating inflammation using corticosteroids, biologics, and other immune-modulating agents, but these therapies often come with limitations, including side effects and insufficient resolution of symptoms [6].

This review aims to systematically analyze the shared inflammatory pathways and biomarkers across various chronic diseases. By highlighting the clinical implications of inflammation-targeted treatments and identifying knowledge gaps, particularly in autoimmune diseases, this review will provide insights into future therapeutic strategies.

## Review

Sources and selection criteria

Between 2011 and 2025, we conducted a thorough search of relevant literature on chronic inflammation across multiple medical specialties. Databases such as PubMed, Embase, OVID, and Google Scholar were searched, with studies restricted to English-language, peer-reviewed articles. We used a combination of key terms including "chronic inflammation," "autoimmune," "infection," "biomarkers," "neuropathy," "rheumatology," "hepatology," "oral health," "bowel syndrome," "hepatitis," and "periodontal disease" and disease-specific terms such as "peripheral neuropathy," "arthritis," and "sepsis." Priority was given to systematic reviews, clinical trials, and narrative reviews, while case reports and retrospective studies were also included. Additionally, the reference lists of selected articles were examined for further relevant studies.

Chronic inflammatory syndrome commonly associated with infectious causes

Chronic inflammatory syndromes induced by infectious agents, both viral and bacterial, have been increasingly recognized for their role in triggering long-lasting immune responses and contributing to various chronic conditions. Infectious agents such as viruses, bacteria, and their respective products often trigger molecular mimicry, bystander activation, and epitope spreading, mechanisms that result in persistent inflammation and chronic pain [7]. Viral infections such as Epstein-Barr virus (EBV), Zika virus, and human immunodeficiency virus (HIV), as well as bacterial infections such as *Campylobacter jejuni *infection, have been linked to inflammatory syndromes that last for months or even years. These chronic conditions often involve neuropathy, arthritis, and other systemic inflammatory responses, leading to debilitating symptoms [8].

Mechanisms of infectious agents in causing inflammatory syndromes

The mechanism by which viral and bacterial infections induce chronic inflammation is complex and multifaceted. Molecular mimicry is one of the primary hypotheses, where pathogens possess antigens that resemble the body's own molecules, leading to an autoimmune response. In Guillain-Barré syndrome (GBS), for example, *Campylobacter jejuni* infection triggers an immune response that attacks the peripheral nervous system by mimicking gangliosides on nerve axolemma [9]. Similarly, the molecular mimicry hypothesis extends to diseases such as multiple sclerosis and reactive arthritis. Studies in rodents have demonstrated that viral infections can elevate pain sensitivity by activating inflammatory pathways. Research in rodents infected with murine leukemia retrovirus (an analogue to HIV) showed an increase in pain sensitivity due to heightened levels of the enzyme indoleamine-2,3-dioxygenase, which remained elevated as the infection persisted [10]. Similarly, studies in humans, especially those living with HIV, have shown that detectable viral loads are linked with increased pain sensitivity, suggesting a direct link between persistent infection and chronic pain [11].

Products produced by viral infections and their role in chronic inflammation

Viral infections, particularly those involving herpesviruses (e.g., Epstein-Barr virus and cytomegalovirus), play a significant role in chronic inflammation through their ability to remain dormant within the host and reactivate when immunity wanes. During reactivation, these viruses can cause neuropathic pain, central sensitization, and a variety of inflammatory syndromes, including myalgic encephalitis or chronic fatigue syndrome (CFS), which are characterized by diffuse chronic pain [12]. HIV has been linked with various neuropathic conditions, including painful peripheral neuropathy, which often manifests as "stocking and glove" distribution. Research has shown that viral components such as glycoprotein 120 of HIV can induce axonal degeneration and inflammatory cytokine release, further contributing to chronic pain. Additionally, viral remnants such as mRNA or proteins can perpetuate immune responses, leading to chronic inflammation and pain (Table 1) [13].

**Table 1:** Overview of Infectious Agents Across Medical Specialties: Presentation and Treatment Approaches HIV: human immunodeficiency virus, SARS-CoV-2: severe acute respiratory syndrome coronavirus 2, NSAIDs: nonsteroidal anti-inflammatory drugs, CFS: chronic fatigue syndrome

Medical Specialty	Disease/Syndrome	Infectious Agents	Presentation	Treatment
Neuropathy/neurophysiology	Neuropathic pain	Viruses: varicella-zoster virus, herpes simplex, HIV, Epstein-Barr virus, cytomegalovirus	Lancinating, shooting pain, numbness, dysesthesias, weakness, often stocking-glove pattern	Antimicrobial treatment (for viral loads), high-concentration capsaicin, acetyl-L-carnitine, neuropathic pain treatment
Resuscitation	Myocarditis	Viruses: coxsackievirus, parvovirus, SARS-CoV-2, Epstein-Barr virus, herpes simplex	Chest pain, heart failure signs, arrhythmias	Antiviral therapy, supportive care, immunosuppressants in severe cases
Hepatology	Hepatitis	Viruses: hepatitis A, B, C, D, E	Jaundice, fatigue, abdominal pain, elevated liver enzymes	Antivirals (e.g., pegylated interferon, direct-acting antivirals), supportive care
Rheumatology	Arthritis/arthropathy	Viruses: parvovirus B19, chikungunya virus, hepatitis B, Epstein-Barr virus	Joint pain, swelling, stiffness, worse with activity	Antiviral therapy, NSAIDs, corticosteroids, immunosuppressants
Oral health	Gingivitis/periodontal disease	Viruses: herpes simplex virus (cold sores)	Painful blisters on gums, lip swelling, gingival redness	Antiviral therapy (acyclovir), topical treatments, good oral hygiene
Chronic disease management	CFS	Viruses: Epstein-Barr virus, cytomegalovirus, parvovirus B19, human herpesvirus 6, SARS-CoV-2	Fatigue, muscle pain, difficulty sleeping, cognitive dysfunction	Antiviral therapy (if acute), antidepressants, supportive care, lifestyle modifications

Treatment options for chronic inflammatory syndromes

Treatment of chronic inflammatory conditions related to infections is challenging and often requires a multifaceted approach. Vaccination, while a critical tool in preventing infections, can also lead to chronic pain due to immune-mediated reactions. Vaccine-induced pain is typically a result of inflammatory cytokines (interleukin-1 and interleukin-6) released following antigen exposure. This immune activation can lead to localized or widespread pain, particularly in the case of vaccines targeting viruses such as influenza or hepatitis B [14]. Additionally, antimicrobial treatments can also induce pain through mechanisms such as axonal toxicity, demyelination, and nerve conduction blockade. Nucleoside analogue reverse transcriptase inhibitors for infections, including those for appendicitis or necrotizing fasciitis, have also been associated with persistent pain, with studies indicating that up to 33% of patients continue to experience pain after gallbladder resection (Table 2) [15].

**Table 2:** Antimicrobial and Antiviral Drug Treatments for Neuropathy and Associated Pain Conditions STDs: sexually transmitted diseases, HIV: human immunodeficiency virus, GI: gastrointestinal

Drug Treatment	Common Indications	Associated Pain Conditions/Symptoms	Comments
Chloramphenicol	Superficial eye/ear infections, typhoid, meningitis	Peripheral neuropathy, bowel inflammation	Rarely used in Europe/North America due to aplastic anemia risk.
Chloroquine/hydroxychloroquine	Malaria, amoebiasis, rheumatic diseases	Peripheral neuropathy, myopathy	Long-term use for rheumatic conditions may cause chronic pain.
Clioquinol	Fungus/protozoan infections, mixed with other agents	Peripheral neuropathy, spinal cord demyelination	Banned in many countries; resurgence for cancer, neurodegenerative studies.
Dapsone	Leprosy, Pneumocystis prophylaxis, dermatitis herpetiformis	Peripheral neuropathy, organ inflammation (nephritis, hepatitis)	Alternative for Pneumocystis prophylaxis in immunocompromised patients.
Fluoroquinolones	Broad-spectrum bacteria, tuberculosis, mycobacterial infections	Peripheral neuropathy, tendinopathy, myopathy, hepatitis	Associated with black box warnings for tendon rupture, neuropathy.
Griseofulvin	Ringworm infections (e.g., athlete’s foot)	Peripheral neuropathy, myopathy, hepatitis	Treatment duration is long, used after topical treatment fails.
Isoniazid	Tuberculosis (active/latent), non-tuberculous mycobacteria	Peripheral neuropathy, myopathy, hepatitis, lupus-like syndrome	Co-treatment with pyridoxine can prevent neuropathy.
Metronidazole	Anaerobic bacteria, parasitic infections (e.g., amoebiasis)	Peripheral neuropathy, oral ulcers, pelvic pain, proctitis	Used for STDs and post-colorectal surgery.
Didanosine	HIV	Peripheral neuropathy, optic neuritis, myopathy, pancreatitis, GI symptoms	Co-treatment with stavudine increases side effects.
Stavudine	HIV	Peripheral neuropathy, pancreatitis, lactic acidosis	Side effects are dose-related and reversible.
Zidovudine	HIV, perinatal HIV transmission prevention	Myopathy, headache, hepatitis	Long treatment courses, side effects reversible.
Nevirapine	HIV	Hepatitis, peripheral neuropathy, gastrointestinal symptoms	Risk of hepatitis with concurrent infections.
Etravirine	HIV	Hepatitis, peripheral neuropathy, GI symptoms, toxic epidermal necrolysis	Treatment courses may last years, incidence hard to pinpoint.
Lamivudine	HIV, hepatitis B	Peripheral neuropathy, pancreatitis, myopathy	Mild neuropathy, usually with other nucleoside reverse transcriptase inhibitors.

Inflammatory pathways and biomarkers in chronic inflammatory diseases across medical specialties

Chronic Fatigue Syndrome (CFS)

Chronic fatigue syndrome, also known as myalgic encephalomyelitis (ME), is characterized by persistent, unexplained fatigue that worsens after minimal exertion. This syndrome is closely linked to various autoimmune and infectious triggers, with studies identifying post-infectious inflammation, particularly after viral infections such as Epstein-Barr virus. The inflammatory pathways in CFS involve cytokines such as TNF-α and interleukin-6 (IL-6), which play key roles in neuroinflammation. Biomarkers such as increased pro-inflammatory cytokines in the cerebrospinal fluid (CSF) are prominent, and recent studies have proposed that this inflammation contributes to the neuroendocrine and autonomic dysfunction seen in CFS patients [16].

Recent research has indicated that infections may trigger autoreactive immune responses in genetically vulnerable individuals, leading to disrupted energy metabolism within the brain and muscles. Distinct immune signatures, including heightened pro-inflammatory cytokine expression, correlate with the severity of ME/CFS symptoms, suggesting a pivotal role of immune-mediated neuroinflammation. Despite interest in antiviral therapies such as acyclovir, valacyclovir, and rintatolimod, their clinical utility remains inconclusive due to inconsistent results and limitations in study design. Likewise, immunomodulatory agents such as corticosteroids, cyclophosphamide, and rituximab have not shown widespread efficacy. These insights highlight the heterogeneous nature of ME/CFS and emphasize the urgent need for tailored, multifaceted treatment approaches within a precision medicine framework [17].

Peripheral Neuropathy

Peripheral neuropathy, commonly linked to conditions such as diabetes and autoimmune disorders, is characterized by persistent pain, tingling, and sensory deficits. The underlying pathophysiology involves immune system dysregulation, which drives inflammation in peripheral nerves through the activation of inflammatory cytokines and increased oxidative stress. Elevated levels of biomarkers such as TNF-α, IL-1β, and nerve growth factor (NGF) are frequently observed in neuropathic conditions, contributing to nerve damage and pain [18]. The global prevalence of HIV, with over 30 million individuals infected, is often underestimated due to limited healthcare access in high-risk populations. Although the advent of antiretroviral therapy (ART) has significantly improved the prognosis of HIV, chronic infection remains a challenge, with over half of HIV-positive individuals experiencing chronic pain, including painful peripheral neuropathy. Older ART regimens have been associated with an increased risk of distal polyneuropathies, likely through inflammatory cytokine upregulation and microglial activation. Furthermore, HIV patients are more susceptible to other pain syndromes, including nociceptive and nociplastic pain, which may arise from central and peripheral sensitization as well as psychosocial stressors such as stigma [19]. In a similar vein, the study of chronic inflammatory demyelinating polyneuropathy (CIDP) and multifocal motor neuropathy (MMN) has revealed significant differences in functional limitations, underscoring the need for tailored rehabilitation. While CIDP patients experience more extensive mobility and exercise restrictions, MMN patients primarily face challenges with fine motor skills. These findings highlight the importance of personalized care strategies and underscore the need for integrated, multidisciplinary approaches that address both the physical and psychosocial aspects of the disease [20].

Sepsis

Sepsis is a life-threatening condition characterized by a dysregulated systemic inflammatory response to infection, leading to extensive tissue injury and multiple organ failure. This immune overactivation is primarily mediated by pro-inflammatory cytokines such as TNF-α, IL-6, and IL-1β. Elevated levels of these cytokines, along with biomarkers such as C-reactive protein (CRP), serve as indicators of disease severity [21]. Recent studies have highlighted the vagus nerve's essential role in regulating immune responses during sepsis. Alterations in heart rate variability, reflecting vagal tone, are now considered predictive of sepsis severity, with reduced vagal activity linked to higher risk of septic shock and death. Experimental data show that the disruption of vagal signaling or α7 nicotinic acetylcholine receptor (α7nAChR) deficiency increases mortality, while the activation of this pathway, either pharmacologically or through nerve stimulation, suppresses cytokine release and enhances survival. As α7nAChR also plays a role in central inflammation, it presents a promising target for novel immunomodulatory therapies. These findings hold significant implications for other immune-mediated disorders, including chronic fatigue syndrome, where similar inflammatory mechanisms may be involved [22].

Post-cardiac Arrest Syndrome (PCAS)

PCAS is a multifaceted condition that arises following the resuscitation of patients after cardiac arrest. It is characterized by systemic inflammation, myocardial dysfunction, and neurological impairment, all of which contribute to poor long-term outcomes. Inflammation plays a central role in the pathophysiology of PCAS, with cytokines such as IL-6 and CRP being elevated in response to the inflammatory reflex. Recent advancements have highlighted the use of biomarkers, such as neurofilament light chain (NFL), to assess the extent of brain injury in these patients. These biomarkers offer valuable prognostic information, allowing for better prediction of neurological recovery and providing insights into the severity of both myocardial and brain injury post-resuscitation [23].

Additionally, there has been growing interest in interventions aimed at modulating the inflammatory response to improve outcomes in PCAS. Vagus nerve stimulation (VNS), which targets the inflammatory reflex, has shown promise in reducing systemic inflammation and enhancing both myocardial and neurological recovery. Furthermore, advanced imaging techniques such as magnetic resonance imaging (MRI) and positron emission tomography (PET) are increasingly being utilized to assess brain injury and inflammation, providing a more comprehensive approach to managing PCAS. These innovations in both diagnostic tools and therapeutic strategies are crucial for improving the prognosis of patients affected by this complex condition [24].

Hepatitis

Chronic viral hepatitis, primarily caused by hepatitis B and C, remains a leading cause of liver disease globally, driven by persistent viral infections that trigger chronic inflammation. This inflammation leads to liver damage, including fibrosis, cirrhosis, and eventually liver failure. Inflammatory pathways are activated through the production of cytokines such as IL-1β, IL-6, and TNF-α, contributing to liver injury. Traditional biomarkers, such as alanine aminotransferase (ALT) and aspartate aminotransferase (AST), are commonly used to assess liver inflammation, while serum HCV RNA levels play a key role in monitoring viral load in hepatitis C patients. Recent advances in hepatology have resulted in more precise diagnostic tools and therapies, improving patient outcomes and helping to prevent disease progression [25].

The advent of direct-acting antiviral (DAA) therapies has transformed hepatitis C management, significantly improving viral clearance and reducing complications such as cirrhosis and hepatocellular carcinoma (HCC). These therapies, including protease inhibitors, polymerase inhibitors, and NS5A inhibitors, have demonstrated high success in curing hepatitis C and lowering viral loads to undetectable levels in most patients. Non-invasive tools such as the Enhanced Liver Fibrosis (ELF) test and transient elastography (FibroScan) now allow accurate assessment of liver fibrosis, minimizing the need for invasive biopsies. Moreover, emerging biomarkers such as serum microRNAs are aiding in early detection and prognosis. Immune checkpoint inhibitors and personalized medicine are further advancing targeted, patient-specific treatment approaches [26].

Rheumatoid Arthritis (RA)

RA is a chronic autoimmune disorder marked by persistent joint inflammation that causes pain, swelling, and progressive joint destruction. The underlying inflammatory process is primarily driven by T-cell activation and the release of pro-inflammatory cytokines such as TNF-α, IL-1, and IL-6. These cytokines promote synovial inflammation and contribute to joint degradation. Diagnostic tools for RA include biomarkers such as rheumatoid factor (RF) and anti-citrullinated protein antibodies (ACPA), which help in both confirming the diagnosis and assessing disease activity [27].

Recent advances in RA research have shed light on the potential of neuromodulation as a therapeutic approach, particularly through the cholinergic anti-inflammatory pathway. Experimental models, such as those using type II collagen to induce arthritis, have shown that the activation of α7 nicotinic acetylcholine receptors (α7nAChRs), for instance, through nicotine, can significantly reduce synovial TNF-α levels, mitigate joint inflammation, and limit histopathological damage. These anti-inflammatory effects are thought to arise from altered macrophage activity, a shift toward Th2 or regulatory T-cell dominance, and the suppression of IL-17 production by Th17 cells. Moreover, VNS has emerged as a promising anti-inflammatory intervention, working through both afferent and efferent pathways to engage β-adrenergic receptors via the vagus nerve-locus coeruleus-synovial network, independently of the spleen and adrenal glands. This evolving understanding challenges the conventional view of antagonism between the sympathetic and parasympathetic systems and suggests a more coordinated, sequential model of neuroimmune regulation, offering new prospects for targeted neuromodulatory therapies in autoimmune joint diseases [28].

Chronic Periodontal Disease

Chronic PD, primarily instigated by microbial imbalance in the oral environment, initiates an immune-inflammatory cascade characterized by the secretion of pro-inflammatory cytokines such as TNF-α, IL-1β, and IL-6. These mediators not only intensify local tissue damage and bone resorption but also contribute to clinical attachment loss (CAL). Pathogens such as *Porphyromonas gingivalis*, *Treponema denticola*, and *Tannerella forsythia* play a central role in both the progression of periodontal damage and the perpetuation of systemic inflammation. Recent insights have revealed that genetic susceptibilities, particularly those associated with inflammatory conditions such as nonalcoholic fatty liver disease, may heighten an individual's risk of developing PD [29]. Furthermore, advancements in biomarker research have identified both microbial and host-derived indicators, including adiponectin, resistin, chemerin, matrix metalloproteinases (MMPs), and salivary exosomal PD-L1 RNA, as critical tools for detecting and assessing disease activity. The integration of high-throughput technologies, such as proteomics and genomic profiling, continues to enhance the precision and reliability of these diagnostic approaches.

In terms of therapeutic progress, the contemporary focus has evolved from traditional anti-inflammatory suppression toward the modulation and resolution of chronic inflammation. Pharmacological interventions utilizing natural compounds such as oleuropein, resolvins, colchicine, and panduratin A, along with statins and emerging agents such as tetramethylpyrazine, have demonstrated potential in mitigating alveolar bone degradation, tissue apoptosis, and overall inflammatory burden in experimental models [30]. Combinatorial antioxidant therapies, notably those incorporating vitamins C and E with lysozyme, have also shown promise in clinical settings for reducing gingival inflammation. These multifaceted advancements reflect a shift toward more holistic, biomarker-guided, and personalized treatment paradigms in managing periodontal inflammation [30].

Role of antimicrobial treatment and bioelectronic medicine in chronic inflammatory diseases

Chronic pain resulting from infections is a complex condition influenced by various factors such as inflammation, central sensitization, tissue damage, and psychological elements. While antimicrobial treatments can effectively reduce pain associated with active infections and inflammation, they offer little relief for chronic pain caused by irreversible tissue damage, nerve injury, or persistent immune responses in the absence of detectable pathogens. Psychological factors such as anxiety, depression, and stress have been found to exacerbate chronic pain, especially following infections such as COVID-19 or HIV. These mental health challenges not only heighten the risk of chronic pain but also contribute to the development of mental health disorders, creating a vicious cycle where pain and psychological distress intensify each other [31]. In the literature search, seven relevant studies were identified that aligned with our review's aim and objectives (Table 3). These studies consistently highlighted that while antibiotics may provide pain relief in certain infection-associated conditions, such as irritable bowel syndrome (IBS), prostatitis, post-surgical pain, and Modic type 1 disc changes, their effectiveness is limited or inconsistent in cases involving chronic pain without active infection or in systemic conditions such as coronary artery disease or functional dyspepsia.

**Table 3:** Summary of Studies on Antibiotic Treatment for Pain Conditions RCTs: randomized controlled trials, SMD: standardized mean difference, IBD: inflammatory bowel disease, MI: myocardial infarction

Author(s) and Year	Study Design	Patient Characteristics	Key Findings
Ford et al. (2018) [32]	Meta-analysis of RCTs	2,845 patients with irritable bowel syndrome	Antibiotics favored over placebo; rifaximin most studied; low-bias studies showed significant benefit (risk ratio: 0.87).
Anothaisintawee et al. (2011) [33]	Systematic review and network meta-analysis	215 patients with chronic prostatitis or pelvic pain	Antibiotics plus α-blockers more effective; pain reduction with a pooled risk ratio of -4.4.
Wanis et al. (2017) [34]	Meta-analysis of RCTs	337 patients post-hemorrhoidectomy	Metronidazole reduced pain postoperatively (SMD: -0.87 to -1.43).
Gilligan et al. (2021) [35]	Narrative review	413 patients with chronic low back pain and vertebral endplate signal changes	Antibiotics (amoxicillin-clavulanic acid) showed pain reduction in positive studies; further research needed.
Norton et al. (2017) [36]	Systematic review	145 patients with IBD	Metronidazole and ciprofloxacin reduced abdominal symptoms with no significant difference between the two.
Swedish Council on Health Technology Assessment (2007) [37]	Systematic review of RCTs and literature reviews (1999-2005)	Patients with functional dyspepsia	Helicobacter pylori eradication offers mild relief; most patients uninfected, limited benefit.
Andraws et al. (2005) [38]	Meta-analysis of RCTs evaluating antichlamydial antibiotics	19,217 patients with coronary artery disease	No significant benefit in reducing angina, MI, or mortality.

Bioelectronic medicine emerges as a promising approach to modulating inflammatory responses by targeting neural pathways. This innovative field uses the vagus nerve and cholinergic pathways to control inflammation through electrical stimulation or specific pharmacological agents. With its ability to provide precise neural modulation, bioelectronic medicine holds substantial potential for treating conditions such as rheumatoid arthritis, inflammatory bowel disease, and depression. Early research has shown that VNS can significantly reduce inflammation and improve clinical outcomes in autoimmune diseases. The goal is to develop devices capable of influencing neural signals to treat chronic inflammation and immune dysfunction [39]. Ongoing studies, including animal models and small clinical trials, suggest promising results. Furthermore, the rise of non-invasive techniques such as transcutaneous cervical vagus nerve stimulation is making this therapy more accessible and patient-friendly. As the field progresses, bioelectronic medicine continues to show potential not only for autoimmune diseases but also for other conditions, including cancer, obesity, and metabolic disorders. The application of electrical stimulation to control inflammation is expanding, and as VNS becomes more refined, it could revolutionize the treatment of chronic inflammatory diseases by harnessing the body's own neural circuits to regulate immune responses [40].

**Figure 1:**
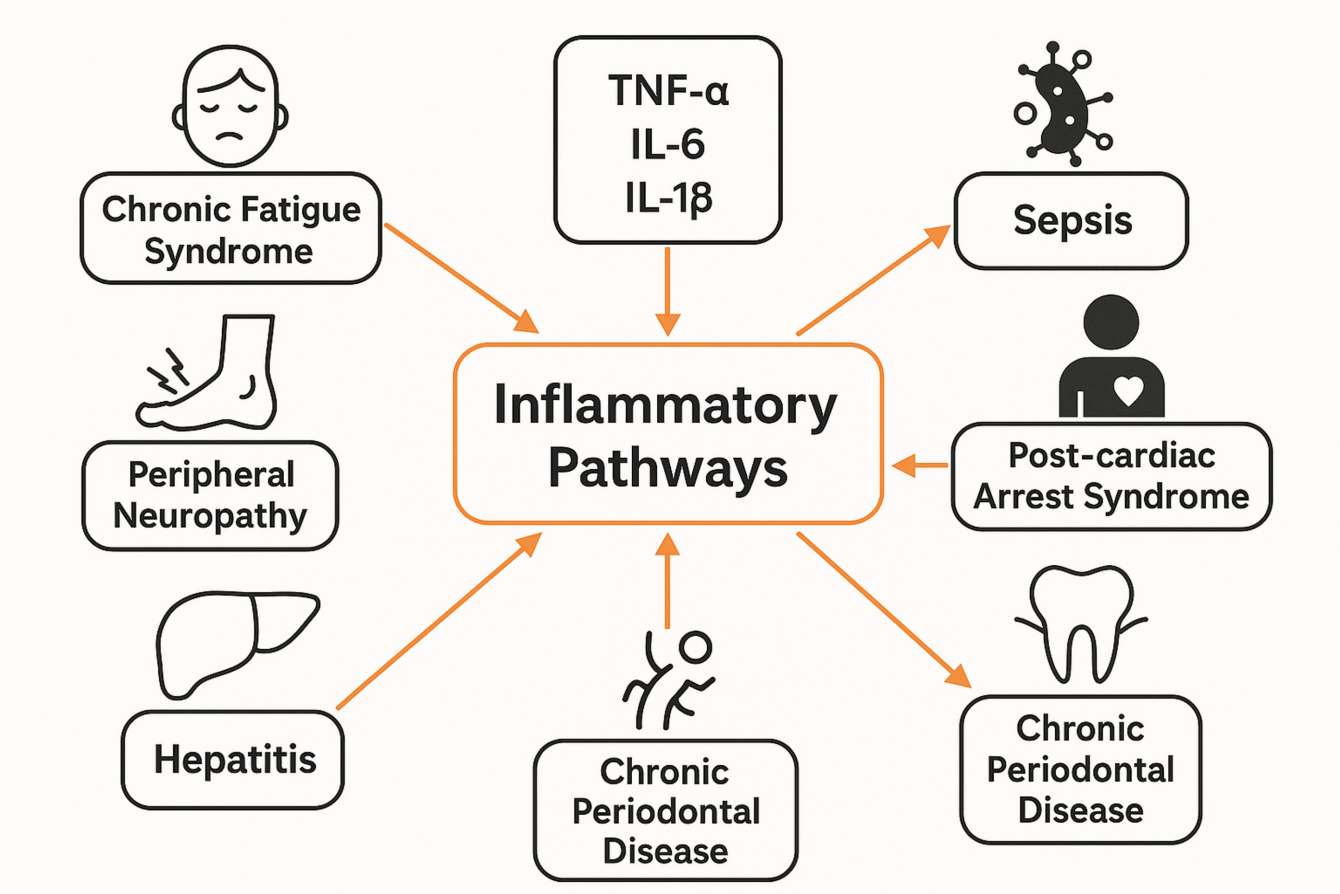
Mechanistic Pathways Linking Infection-Induced Inflammation to Chronic Pain and Neurological Dysfunction TNF-α: tumor necrosis factor-alpha, IL-1β: interleukin-1 beta, IL-6: interleukin-6

Figure 1 presents the mechanistic pathways linking infection-induced inflammation to chronic pain and neurological dysfunction.

The shortcomings of this study include restricting the literature search to the 2011-2025 period and English-language articles, which may introduce selection bias. Future reviews might consider non-English studies, which could broaden the scope of evidence.

## Conclusions

Chronic pain arising from infectious etiologies represents a multifaceted clinical challenge, underpinned by persistent inflammation, immune dysregulation, and tissue injury. Both viral and bacterial infections initiate cascades of inflammatory signaling, through cytokine release, microglial activation, and immune cell infiltration, that perpetuate pain and contribute to long-term complications such as neuropathy and systemic dysfunction. Although significant progress has been made in elucidating these pathophysiological mechanisms, the translation of these insights into effective, personalized treatments remains in its infancy. To bridge the gap between mechanistic understanding and clinical intervention, several translational pathways warrant immediate exploration. Pilot studies investigating bioelectronic medicine, such as vagus nerve stimulation or closed-loop neuromodulation, hold promise in modulating neuroimmune circuits and attenuating chronic inflammation. These interventions, grounded in the emerging field of electroceuticals, may offer non-pharmacologic, precision-based strategies for pain control and immune regulation.

Additionally, the development and clinical validation of emerging biomarkers, including microRNAs, metabolomic profiles, and cytokine panels, could enable real-time monitoring of inflammatory states and disease progression. These dynamic biomarkers have the potential to stratify patients, predict treatment response, and tailor therapeutic regimens, paving the way for precision medicine in chronic inflammatory pain syndromes. Future research should also prioritize integrative management strategies that address the biopsychosocial dimensions of chronic pain. This includes embedding lifestyle interventions, such as anti-inflammatory diets, physical activity, stress reduction techniques, and sleep optimization, into clinical care pathways. Holistic approaches, when combined with pharmacological and interventional therapies, may amplify treatment efficacy and improve quality of life for affected individuals.
